# Myeloid cell deletion of Aryl hydrocarbon Receptor Nuclear Translocator (ARNT) induces non-alcoholic steatohepatitis

**DOI:** 10.1371/journal.pone.0225332

**Published:** 2019-12-04

**Authors:** Christopher Scott, Rebecca Stokes, Kuan Minn Cha, Andrew Clouston, Mohammed Eslam, Mayda Metwally, Michael M. Swarbrick, Jacob George, Jenny E. Gunton

**Affiliations:** 1 Centre for Diabetes, Obesity and Endocrinology, The Westmead Institute for Medical Research, The University of Sydney, Sydney, NSW, Australia; 2 Sydney Medical School, The University of Sydney, Sydney, NSW, Australia; 3 Garvan Institute of Medical Research, Darlinghurst, NSW, Australia; 4 Envoi Specialist Pathologists, Brisbane, Queensland, Australia; 5 Storr Liver Centre, The Westmead Institute for Medical Research, The University of Sydney, Sydney, NSW, Australia; 6 St. Vincent’s Clinical School, University of NSW, Sydney, NSW, Australia; Universidade do Estado do Rio de Janeiro, BRAZIL

## Abstract

**Background and aim:**

Non-alcoholic steatohepatitis (NASH) is predicted to become the most common cause of cirrhosis and liver failure. Risk factors include obesity, insulin resistance and diabetes. Macrophages and other myeloid cells play crucial roles in initiating and driving inflammation. Aryl hydrocarbon Receptor Nuclear Translocator (ARNT) is a transcription factor which binds to a range of partners to mediate responses to environmental signals, including the diet. In people with diabetes it is decreased in liver. We hypothesised that myeloid cell ARNT activity may contribute to the development of liver pathology.

**Methods:**

Floxed-ARNT mice were bred with LysM-Cre mice to generate mice with reduced ARNT in myeloid cells. Animals were fed a high fat diet (HFD) and liver pathology was assessed. Histology, mRNA, fat accumulation and metabolism were studied.

**Results:**

Animals with reduced myeloid ARNT developed steatohepatitis on a HFD, with additional alterations of metabolism and fat deposition. Steatohepatitis was accompanied by hepatic macrophage infiltration and expression of both M1 and M2 markers. Expression of mRNAs for *Cxcl1*, *Mcp-1*, *Tnf-α* and *Tgf-β1* were increased. Human livers from controls and people with NASH were tested; ARNT mRNA was decreased by 80% (p = 0.0004).

**Conclusions:**

Decreased myeloid ARNT may play a role in the conversion from non-alcoholic fatty liver to steatohepatitis. Increasing ARNT may be a therapeutic strategy to reduce NASH.

**Highlights**

The determinants of conversion from benign fatty liver disease to non-alcoholic steatohepatitis are not well understood. These studies show that lack of the transcription factor ARNT in myeloid cells predisposes mice to NASH.

Type 2 diabetes (T2D) and liver disease are commonly associated. Liver ARNT is decreased in people with T2D and with liver disease. ARNT may be a common pathogenic factor in diabetes and liver disease.

## Introduction

Non-alcoholic fatty liver disease (NAFLD) is defined as the accumulation of excess, microscopically visible lipid in hepatocytes in the absence of excessive alcohol consumption and is now the most common chronic liver disease in developed countries [1, 2]. The risk of NAFLD is increased with obesity, insulin resistance and type 2 diabetes mellitus (T2DM); the incidence of these risk factors has been increasing in recent years [3].

Amongst overweight and obese people with type 2 diabetes, NAFLD is present in over 50% of cases. Importantly, ~20% of patients with NAFLD progress to develop non-alcoholic steatohepatitis (NASH), which in addition to steatosis is characterised by lobular inflammation, hepatocyte ballooning and fibrosis [4]. NAFLD is relatively benign, however, NASH increases the risk of cirrhosis, liver failure and hepatocellular cancer [5, 6], with rates of cirrhosis estimated at 5–20% over 10 years [1]. Factors known to influence the progression from NAFLD to NASH include greater hepatocyte lipid accumulation, insulin resistance, oxidative stress leading to lipid peroxidation, production of pro-inflammatory cytokines, and mitochondrial dysfunction [7–9]. NASH also occurs in the setting of altered systemic concentrations of adipokines (such as leptin, interleukin-6 and adiponectin) which influence hepatic lipid accumulation and insulin sensitivity.

Myeloid cells play a key role in NASH progression. In the liver, macrophages contribute to the development of NASH and fibrosis in both the inflammatory and resolution phases [10–12]. In obesity, excess M1 type macrophages not only accumulate in adipose tissue [13–15] but also in liver [16], where they produce the chemokine (C-C motif) ligand 2/monocyte chemotactic protein-1 (CCL2/MCP-1) [17]. Macrophages also influence whole-body insulin sensitivity and glucose metabolism, as demonstrated by the striking phenotypes of a number of myeloid- and macrophage-specific conditional knockout mice [18–21].

Chronic intermittent hypoxia also contributes to hyperlipidaemia, lipid peroxidation [22] and the development of NASH [23] in mouse models. This relationship is supported by cross-sectional studies in humans with obstructive sleep apnoea [24]. The transcriptional response to hypoxia is regulated by hypoxia-inducible factors (HIFs), active heterodimeric transcriptional complexes that can respond to a variety of environmental signals [25–27]. HIF1 is a heterodimer containing HIF-1α and the Aryl hydrocarbon Receptor Nuclear Translocator (ARNT, also known as HIF-1β); while HIF-2α and ARNT comprise the HIF-2 complex. HIF-1α activity is reduced at high glucose concentrations in human fibroblasts and diabetic animals [28–31]. ARNT with the aryl hydrocarbon receptor (AhR) regulates response to environmental toxins including dioxin and other cyclic hydrocarbons.

Accordingly, deletion of HIFs in myeloid cells decreases NASH progression while myeloid cell-specific HIF-1α or HIF-2α deletion impairs immune function. In contrast, deletion of macrophage AhR leads to opposing effects with an increased acute inflammatory response [32–37]. With regard to ARNT, expression decreases in the liver and pancreatic islets of patients with type 2 diabetes, and deletion of ARNT in these tissues results in impairment of metabolism [25, 38]. We therefore hypothesised that deletion of myeloid ARNT, which binds to HIF-2α, AhR and SIM2 as well as HIF-1α [39], would influence steatohepatitis progression during high-fat diet (HFD) feeding.

## Materials and methods

### Animal studies

All animals received humane care according to the criteria outlined in the “*Australian code of practice for the care and use of animals for scientific purposes*”. Procedures were approved by the Garvan/St. Vincent’s Hospital Animal Ethics Committee. Floxed ARNT mice were created as previously described [25] and bred with LysM-Cre mice [40], to produce myeloid cell ARNT knockout mice (LAR) and floxed-control (FC) littermates. All mice were maintained on an inbred C57Bl/6 background for at least 12 generations.

### Housing and high-fat diet feeding

Mice were housed with a 12-hour light/dark cycle at room temperature, with *ad libitum* access to food and water. From 10–12 weeks of age, mice were fed a high-fat diet (HFD, 45% of energy from fat, based on D12451, Research Diets, New Brunswick, NJ, USA) for 20 weeks until the conclusion of the experiment.

### Metabolic testing

For glucose tolerance tests (GTT) and insulin tolerance tests (ITT), mice were fasted for 6 hours then dextrose (2g/kg body weight) or insulin (0.25U/kg) were given by intraperitoneal (IP) injection. For the pyruvate tolerance tests (PTT), mice were fasted overnight (16 hours) prior to administration of pyruvate (2g/kg, *i*.*p*.). In all tests, blood glucose measurements were taken from tail blood using an Optium glucometer (Abbot Diabetes Care, Doncaster, Australia).

### Tissue collection

Mice were sacrificed after a 6 hour fast at least 1 week after the last metabolic test. Under anaesthesia (2,2,2-tribromoethanol or ketamine/xylazine), blood was collected by cardiac puncture into tubes containing 20 μl of 0.5M EDTA. Plasma supernatant was stored at -80°C. Livers were collected and divided for formalin fixation (histology) and the remainder was snap-frozen in liquid nitrogen for gene expression and lipid studies. Epigonadal and subcutaneous fat depots were collected, weighed and formalin-fixed prior to histology.

### Macrophage isolation

Four days prior to sacrifice, mice were injected with 2ml of 3% thioglycollate IP (Difco, Melbourne, Australia). At sacrifice, after anaesthesia (isofluorane, 5% induction and 2% maintenance), macrophages were isolated by IP injection of 10ml of sterile ice cold PBS. Cells were cultured in RPMI medium (Gibco, Melbourne, Australia) with 10% fetal calf serum and 1% L-glutamine. Two hours later, cells were washed twice with PBS and cultured for a further 24 hours before RNA extraction.

### Gene expression analysis

Samples were lysed in RLT buffer (Qiagen, USA). RNA was isolated and cDNA was synthesized as previously described [41]. Real-time PCR was performed using an ABI7900 instrument in combination with SYBR Green PCR master mix (both from Applied Biosystems, Melbourne Australia). Sequences of PCR primers are available on request. For each gene, mRNA expression was corrected to that of TATA-box binding-protein (*Tbp*) using the 2^ΔΔCT^ method.

As described previously [42] expression of 86 genes related to fatty liver was measured using Mouse Fatty Liver RT^2^ Profiler PCR Arrays (#330231 PAMM-157ZA) and RT^2^ SYBR Green qPCR Mastermix (Qiagen, Doncaster, VIC, Australia). Results were analysed using RT^2^ Profiler software, and expression was normalised to *B2m* (beta-2 macroglubulin), *Gapdh* (glyceraldehyde-3-phosphate dehydrogenase) and *Gusb* (beta glucuronidase) as housekeeping genes.

### Histology

NAFLD / NASH grade was scored by A.C. masked to genotype according to Kleiner *et al* 2005 [43]. Tissues were fixed in 10% buffered formalin, paraffin-embedded and cut into 5μm sections. Sections were stained with haemotoxylin and eosin (H&E), Perl’s stain, Sirius Red or Milligan’s Trichrome staining according to standard protocols. F4/80 staining was performed using the DakoCytomation EnVision+ Dual Link System-HRP (DAB+) Kit (Dako, North Sydney, NSW, Australia) as per manufacturer’s instructions. Following antigen retrieval, rat F4/80 monoclonal antibody was diluted 1:100 in Antibody Diluent (Dako). After staining, slides were counterstained using a standard Haematoxylin protocol. Adipose fat cell size was calculated using ImageJ software by tracing all adipocytes within the microscope field of view and calculating the average size.

### Liver function and triglyceride (TG) content

Plasma Alanine transaminase (ALT) and Aspartate transaminase (AST) levels were measured by the St Vincent’s Hospital Pathology department. Liver TG was extracted from 30-40mg of homogenized liver using a modified Folch method, and measured using a Roche TG kit (GPO-PAP, Mannheim, Germany).

### Western blotting

Fifty mg of snap-frozen liver tissue was homogenised in ice-cold RIPA buffer containing 1 mg/l aprotinin, 1 mg/l leupeptin, 10 mmol/l NaF, 1 mmol/l Na3VO4 and 1 mmol/l PMSF. Cleared lysates were electrophoresed in 12% polyacrylamide gels and transferred to PVDF membranes (Thermo Scientific, #88518). Primary antibodies used included those directed against ADIPOR2 (Sigma SAB1102579), FABP1 (Thermo Scientific, #720242), and MAPK8/JNK (Cell Signaling, #9252S). For mitochondrial oxidative phosphorylation components, we used Total OXPHOS Rodent WB Antibody Cocktail (Abcam, ab110413). Membranes were washed and probed with appropriate HRP-conjugated secondary antibodies (Bio-Rad, Cell Signaling), and proteins were visualised using Super Signal West Pico Chemiluminscent Substrate (Thermo) and a ChemiDoc Imaging System (Bio-Rad). For a loading control, we stripped the membranes with 0.2 M NaOH for 10 minutes and reprobed with an antibody directed against the regulatory molecule (14-3-3 Santa Cruz Biotechnology, catalog #sc-1657). Densitometry was performed using ImageJ software (NIH freeware), and comparisons between FC and LAR livers were performed using unpaired two-tailed t-tests.

### Human liver samples

Human liver samples were obtained from patients with NAFLD who had undergone liver biopsy at Westmead Hospital and had stored liver tissue. Patient characteristics are shown in Fig 7B for this group.

Non-steatotic (control) liver samples were collected from healthy regions of the livers from donors who had undergone liver resection for benign liver tumours for whom all other causes of liver disease were excluded, as previously described [44]. The ethics approval for the normal liver de-identifies the subjects and therefore patient information is not available for the normal group.

Liver biopsies were scored by an expert liver pathologist unaware of clinical data. Histological scoring was based on the system proposed by Kleiner et al. [43]. Steatosis was graded from 0 to 3, lobular inflammation from 0 to 3 and hepatocellular ballooning from 0 to 2. Fibrosis was staged from 0 to 4 with 4 representing cirrhosis. The NAFLD Activity Score (NAS) was calculated to quantify disease activity [43]. All biopsies were of appropriate size and included enough portal tracts for pathological grading and staging of the histological features. Samples with a NAS ≥ 5 or a NAS of 3–4 but with fibrosis were included in the NASH group.

Subjects with evidence of secondary causes of steatosis or alternative diagnoses were excluded including alcohol (men, >30 g/day; women, >20 g/day), total parenteral nutrition, chronic viral hepatitis (hepatitis B and hepatitis C), autoimmune liver diseases, hereditary hemochromatosis, α1-antitrypsin deficiency, Wilson’s disease and drug-induced liver injury. Ethics approval was obtained from the Human Research Ethics Committees of the Sydney West Local Health District and the University of Sydney. Written informed consent was obtained from all participants.

PCR of human liver was done as above, except that RNA from biopsies was assessed using the Agilent 2100 Bioanalyser (Agilent, Waldbronn, Germany) before use. cDNA was prepared using qscript (Quanta Biosciences, Gaithersburg, MD, USA) in a Mastercycler gradient 5331 (Eppendorf AG, Hamburg, Germany). Gene expression for ARNT was measured by qPCR as above. GAPDH was used as the house-keeping gene.

### Statistical analysis

Results were analysed using GraphPad Prism software. LAR and FC mice were compared using two-tailed Student’s t-tests, or nonparametric Kruskal-Wallis Test, as appropriate. Time-course experiments were analysed using repeated measures ANOVA (rmANOVA). A p-value <0.05 was considered significant. Unless indicated otherwise, all data is shown as mean ± SEM.

## Results

### Deletion of ARNT from myeloid cells leads to decreased liver weight and increased subcutaneous fat mass

**Fig 1:**
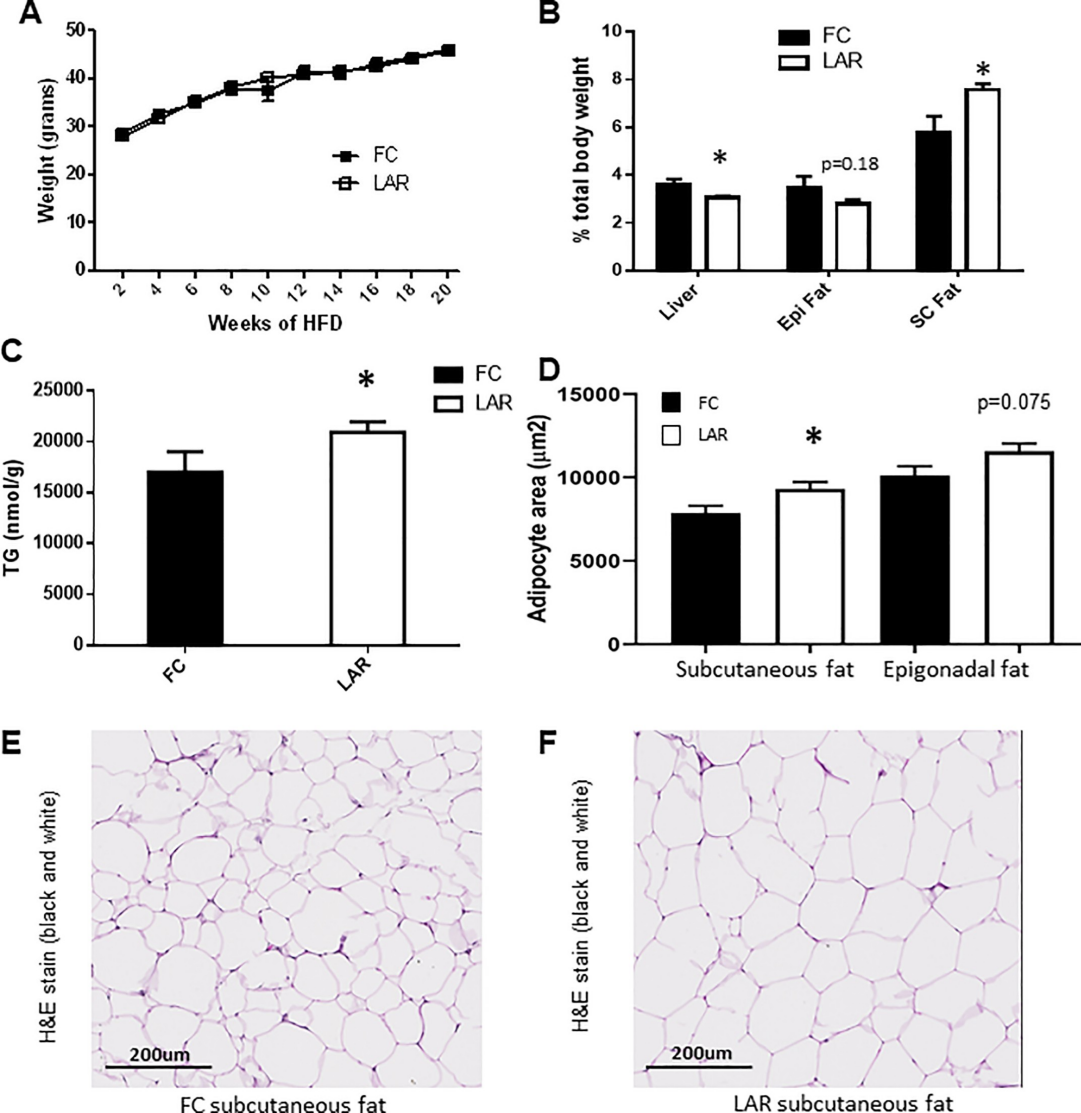
Weight and tissue composition after 20 weeks of high fat diet (HFD). (A) Weight of mice during HFD (n = 14-16/group) did not differ, (B) Tissue weights at sacrifice after 20 weeks of HFD (n = 13-15/group). (C) Liver triglyceride content, measured in duplicate per mouse, n = 5 FC and n = 7 LAR. (D) Quantification of adipocyte size from epigonadal and subcutaneous fat. Representative Hematoxylin and Eosin (H&E) stains of subcutaneous (SC) fat from FC (E) and LAR mice (F) (n = 5-7/group). Epi = epigonadal and SC = subcutaneous inguinal fat. * p<0.05.

ARNT deletion efficiency in this line is >80%, as previously reported [45]. To increase hepatic lipid load, LAR and FC mice were fed a high-fat diet from 10–12 weeks of age (HFD) and continued for 20 weeks. LAR and FC mice had equivalent weight gain (**Fig 1A,** males). However, by 20 weeks feeding, liver weight was reduced in LAR compared to FC mice (1.42g versus 1.71g respectively, or 3.1% versus 3.6% of total body weight, **Fig 1B,** p = 0.046). This was despite a 23% increase in liver triglyceride content (**Fig 1C**).

At sacrifice, despite similar body weight, LAR mice had significantly more subcutaneous fat (3.6±0.2g versus 2.7±0.4g respectively), which equated to 7.6% versus 5.8% of total body weight (p = 0.038, **Fig 1B**). This was accompanied by a significant increase in adipocyte size in this depot (**Fig 1D–1F**). In contrast, overall epigonadal fat mass was not increased (**Fig 1B**) with only a trend to increased mass in LAR mice in this depot (**Fig 1E**, p = 0.075).

### Deletion of ARNT from myeloid cells in mice increases the prevalence of NASH

**Fig 2:**
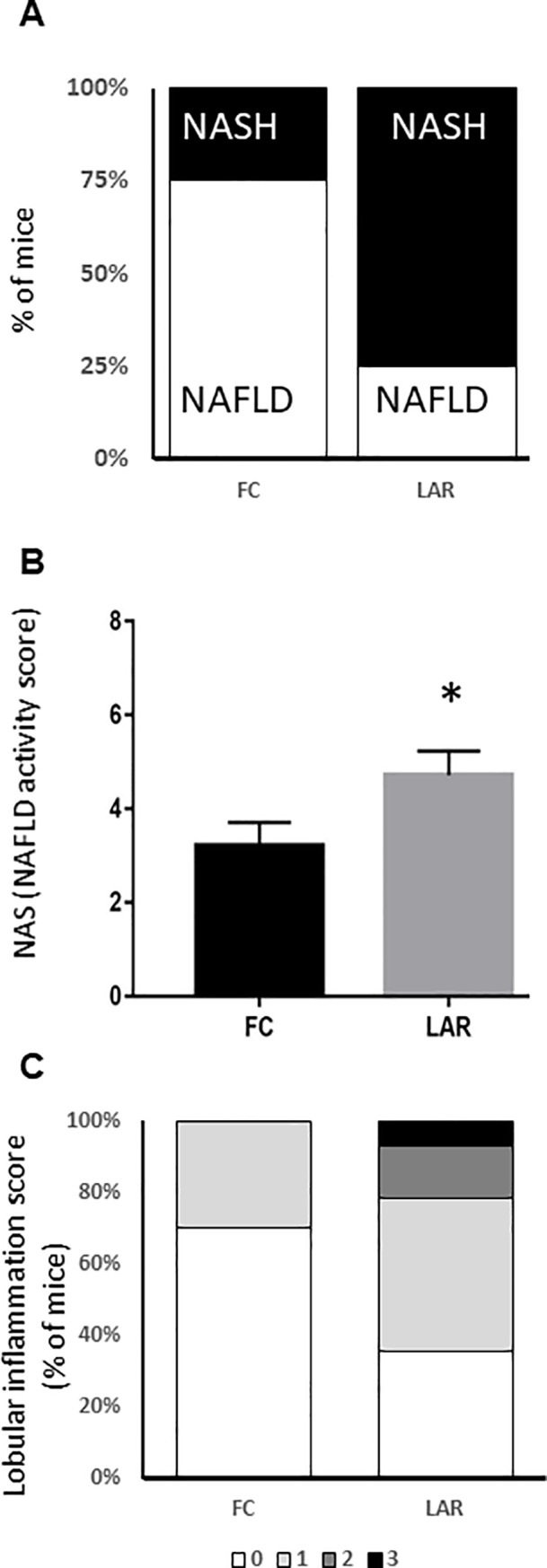
Liver histological scores in control and LAR mice. (A) LAR mice had an increased prevalence of NASH after 20 weeks of high fat feeding. (B) NAFLD activity score (NAS) was significantly higher in LAR mice than FC mice. (C) Lobular inflammation score (a component of NAS) was greater in LAR mice than in controls. * p<0.05.

NASH was scored histologically in HFD-fed LAR and FC mice by a histo-pathologist masked to mouse genotype (A.C.). All mice, regardless of genotype, developed either NAFLD or steatohepatitis at 20 weeks of HFD. Steatohepatitis developed in 9 of 14 LAR mice (64%) versus 3 of 10 FC (30%), **Fig 2A**. Consistent with this, the NAS (NAFLD activity score) was higher in LAR mice at 4.7±0.5 versus 3.2±0.5, p<0.05 (**Fig 2B)**. Individual components of the NAS score showed a trend to greater steatosis (2.7 versus 2.2, p = 0.09), no difference in hepatocyte ballooning (1.1 versus 0.7, p>0.2) and a significant increase in lobular inflammation (0.9±0.3 versus 0.3±0.2, p<0.05, **Fig 2C).**

**Fig 3:**
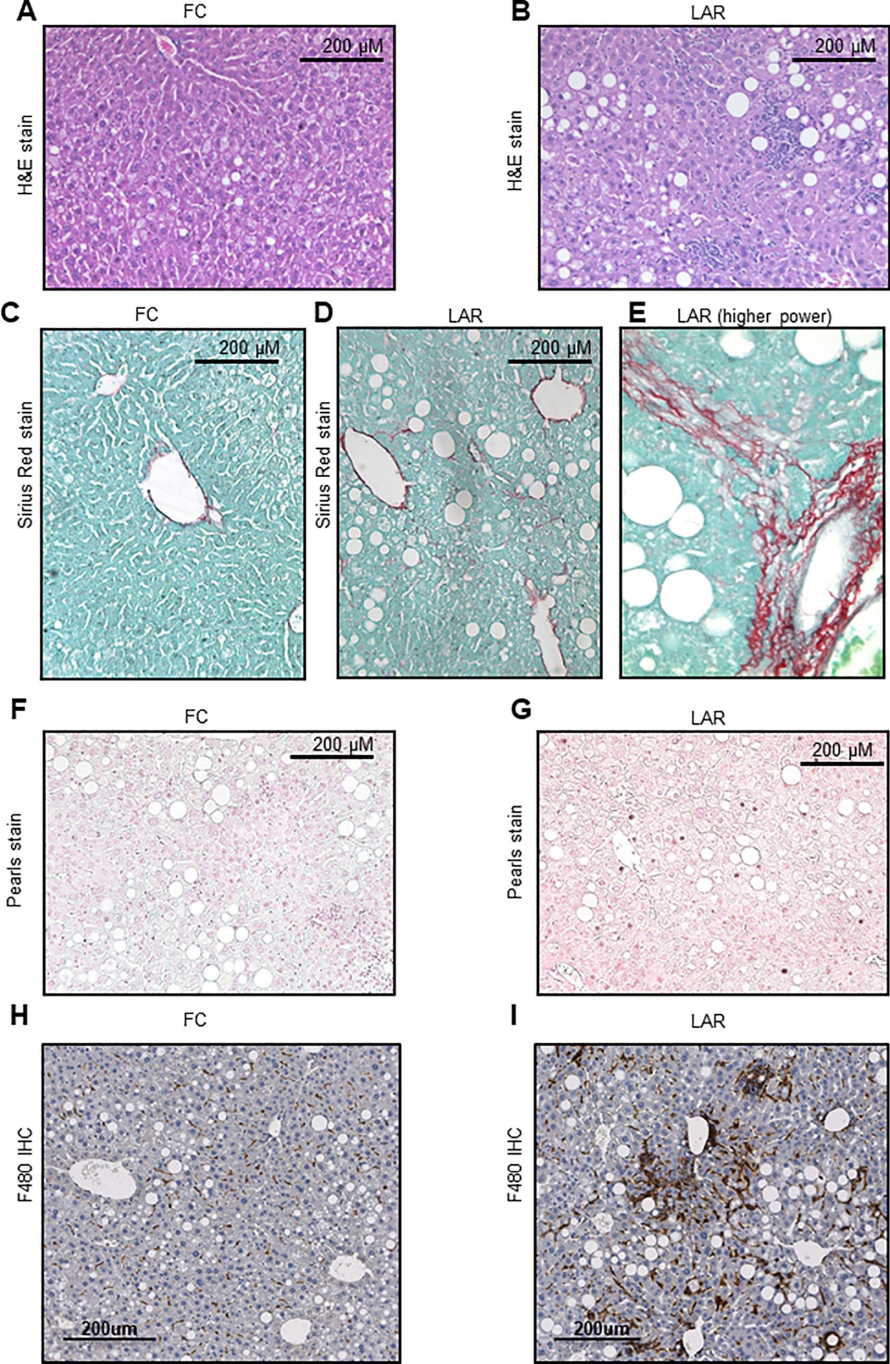
Liver histology after 20 weeks of high fat diet. Hematoxylin and Eosin (H&E) staining of FC (A) and LAR (B) livers. Note the increased steatosis and inflammation in the LAR section. (C) Sirius red staining for collagen in FC mouse. (D) Sirius red in a LAR mouse (E) and higher power image of Sirius red staining in a LAR mouse. Perl’s stain indicated no obvious differences in liver iron content (F) and (G). Macrophage infiltration of the liver was greater in LAR than FC mice, as assessed by F4/80 positive cells (H) and (I).

Representative histological sections for FC and LAR mice are shown in **Fig 3**. Livers from LAR mice had greater TG accumulation than those from FC mice **(Fig 3A–3E)**. Global Sirius red staining to assess fibrosis did not show a significant difference. However, LAR mice had localised areas of increased fibrosis. **Fig 3E** shows a higher-power example of localised fibrosis in a LAR mouse. There was no difference in iron status (Perl’s stain, **Fig 3F and 3G).** Increased macrophage infiltration in the livers of LAR mice was indicated histologically by F4/80 staining (**Fig 3H and 3I)**.

### Changes in gene expression in livers from LAR mice

**Fig 4:**
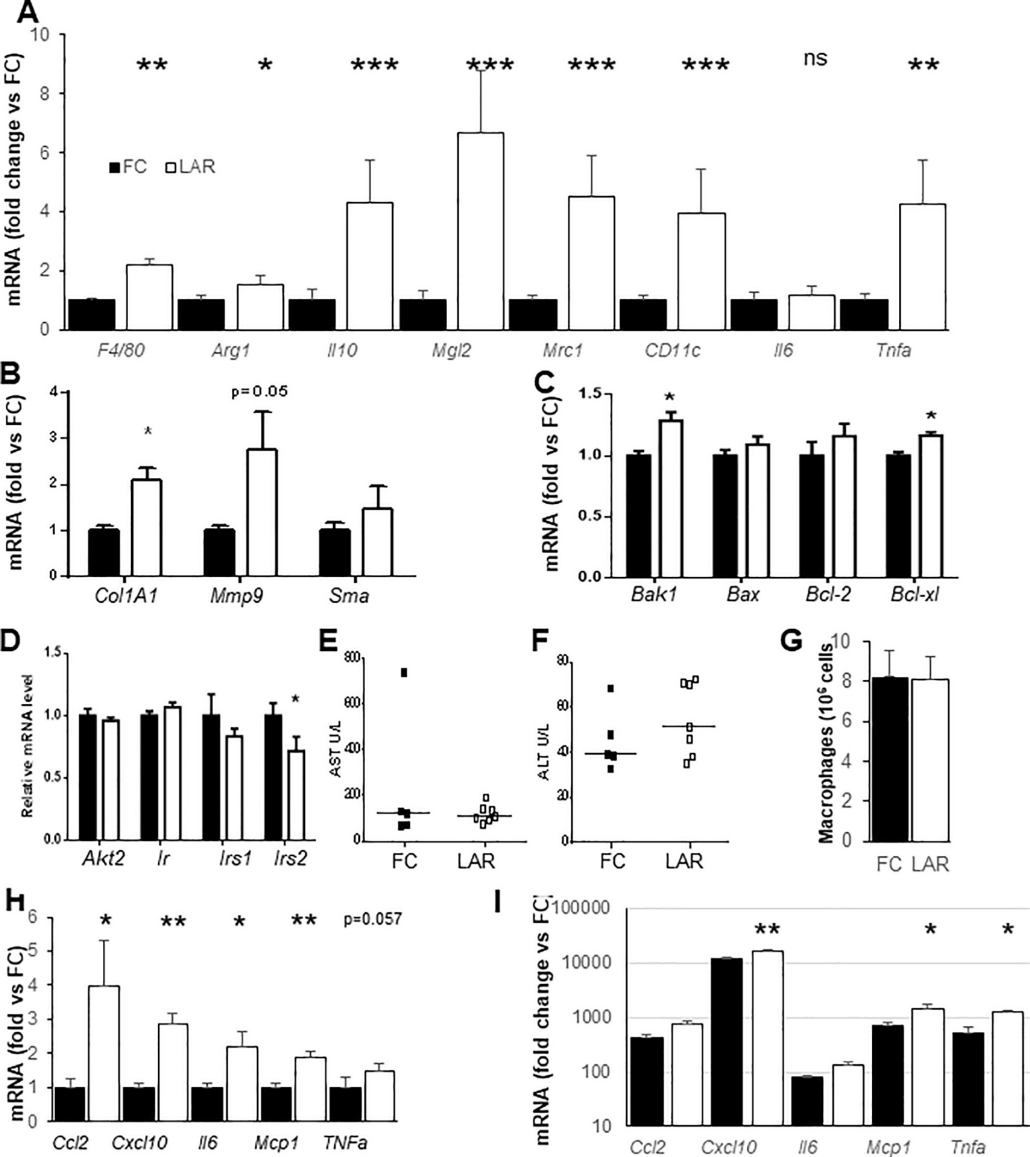
Hepatic gene expression and liver function, and macrophage gene expression. (A) LAR mice had increased mRNA expression of macrophage and inflammation markers. (B) Expression of pro-fibrosis markers including collagen 1A1 were increased in LAR mice. (C) Expression of anti- and pro-apoptotic genes in liver. (D) Expression of insulin signalling components in the liver. (E) Serum aspartate aminotransferase (AsT) levels and (F) Serum alanine aminotransferase (AlT) levels were not significantly different. (G) Peritoneal macrophage numbers from LAR and FC mice. (H) Basal macrophage gene expression and (I) LPS stimulated gene expression in macrophages as fold change versus basal control. Monocyte Chemotactic Protein-1(*Mcp-1*), Chemokine (C-X-C motif) Ligand 1 (*Cxcl1*), Interleukin 6 (*Il-6*), Tumour necrosis factor alpha (*Tnf-α*), Transforming Growth Factor-β1(*Tgf-β1)*, Interleukin 10 (*Il-10*), Alanine transaminase (ALT), Aspartate transaminase (AST), Collagen Type 1α1(*Col1a1*), Matrix Metallopeptidase 9 (*Mmp9)*, Alpha smooth muscle actin (*Sma*), BCL2-Antagonist/Killer 1 (*Bak1*), Bcl-2-associated X protein (*Bax*), B-cell lymphoma 2 (Bcl-2), B-cell lymphoma-extra-large (*Bcl-xl*). * p<0.05, ** p<0.01, *** p<0.001. + p between 0.05 and 0.1. Columns indicate mean and SEM.

Consistent with the histological findings, liver mRNA expression of the macrophage marker *F4/80* was more than two fold higher in LAR HFD mice (**Fig 4A,** p<0.00001). Examination of genes reported to differentiate between M1 and M2 macrophage phenotype showed that both groups were elevated without any obvious preponderance of M1/M2 (**Fig 4A**).

Among pro-fibrogenic genes, there was a significant increase in collagen type 1α1 (*Col1A1* mRNA) and a trend to increased matrix metallopeptidase 9 (*Mmp9)* expression in LAR livers (**Fig 4B**, p<0.0003 and p = 0.052 respectively). In addition, there was increased expression of both Bcl2-antagonist/killer 1 (*Bak1)* and B-cell lymphoma-extra-large *(Bcl-xl)*, (**Fig 4C**, p = 0.0015 and p<0.0005 respectively).

After HFD there was no difference in liver expression of the insulin-signaling genes *Akt2*, insulin receptor *(Ir)*, or insulin receptor substrate 1(*Irs-1)*. There was however a significant decrease in insulin receptor substrate 2 *(Irs-2)* which has been linked to loss of ARNT signalling in other tissues (**Fig 4D**, p = 0.036).

Although steatosis with lobular inflammation was evident histologically there was no significant difference in serum aspartate transaminase (AST) or alanine transaminase (ALT), with wide variability between mice (**Fig 4E and 4F**).

Macrophages were isolated from mice using thioglycollate. Similar numbers were isolated from FC and LAR mice (**Fig 4G**). LAR macrophages had increased basal expression of *Ccl2*, *Cxcl10*, *Il6*, *Mcp1* and a trend to increased *Tnfα* (p = 0.057, **Fig 4H**). Four hours after treatment with LPS, LAR macrophages had significantly greater expression of *Cxcl10*, *Mcp1* and *Tnfα* **(Fig 4I).**

**Fig 5:**
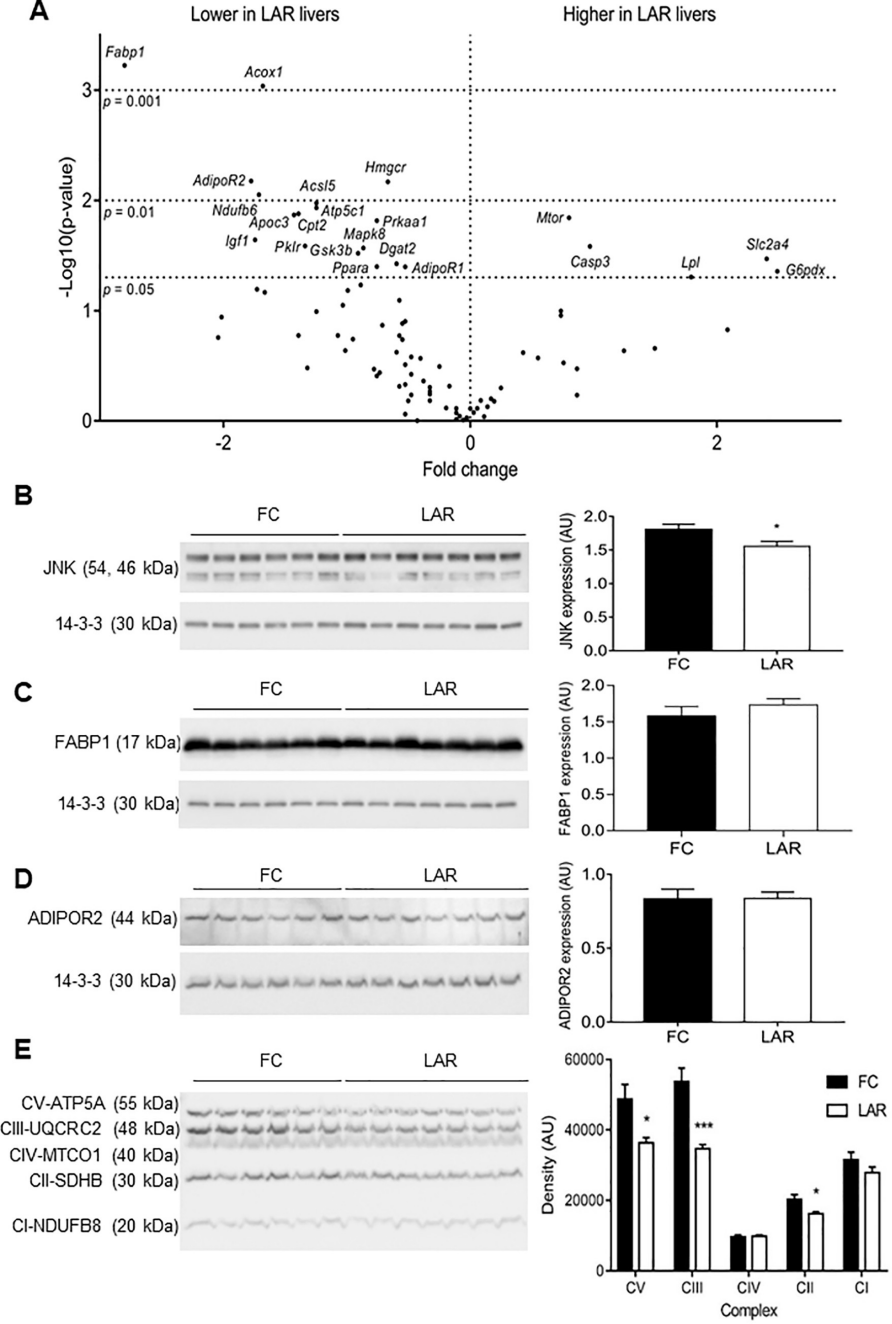
A) Volcano plot of gene expression by PCR array. The gene name is included in each case where expression was significantly altered (p<0.05). Western immunoblots and quantitations for JNK (B), FABP1 (C), and ADIPOR2 (D). Mitochondrial complexes were measured using an antibody cocktail in Fig 5E. Bars indicate mean and SEM. * = p<0.05, *** = p<0.001.

Next, we examined changes in gene expression in livers from LAR and FC mice using PCR arrays (**Fig 5A**). Significantly altered genes are named on the figure. Together these changes in gene expression would contribute to impaired metabolism of lipid, as seen in the livers of the LAR mice.

Protein levels of some of the genes were assessed by Western immunoblotting. Expression of the smaller (46kDa) JNK isoform was decreased, giving an overall decrease in total JNK in LAR livers (p<0.05, **Fig 5B**). Despite significant changes on the PCR arrays, FABP1 and Adiponectin receptor 2 (AdipoR2) protein levels did not differ between groups (**Fig 5C and 5D)**. Disrupted mitochondrial function is proposed to play a role in development of steatohepatitis, so the mitochondrial complexes were assessed as shown in **Fig 5E.** There was a significant decrease in protein levels for Complexes II, III and V (SDHB, UQCRC2 and ATP5A respectively).

Taken together, these results suggest that the increased TG content in LAR livers compared to FC livers may result from impaired fatty acid oxidation.

### LAR mice and glucose homeostasis

**Fig 6:**
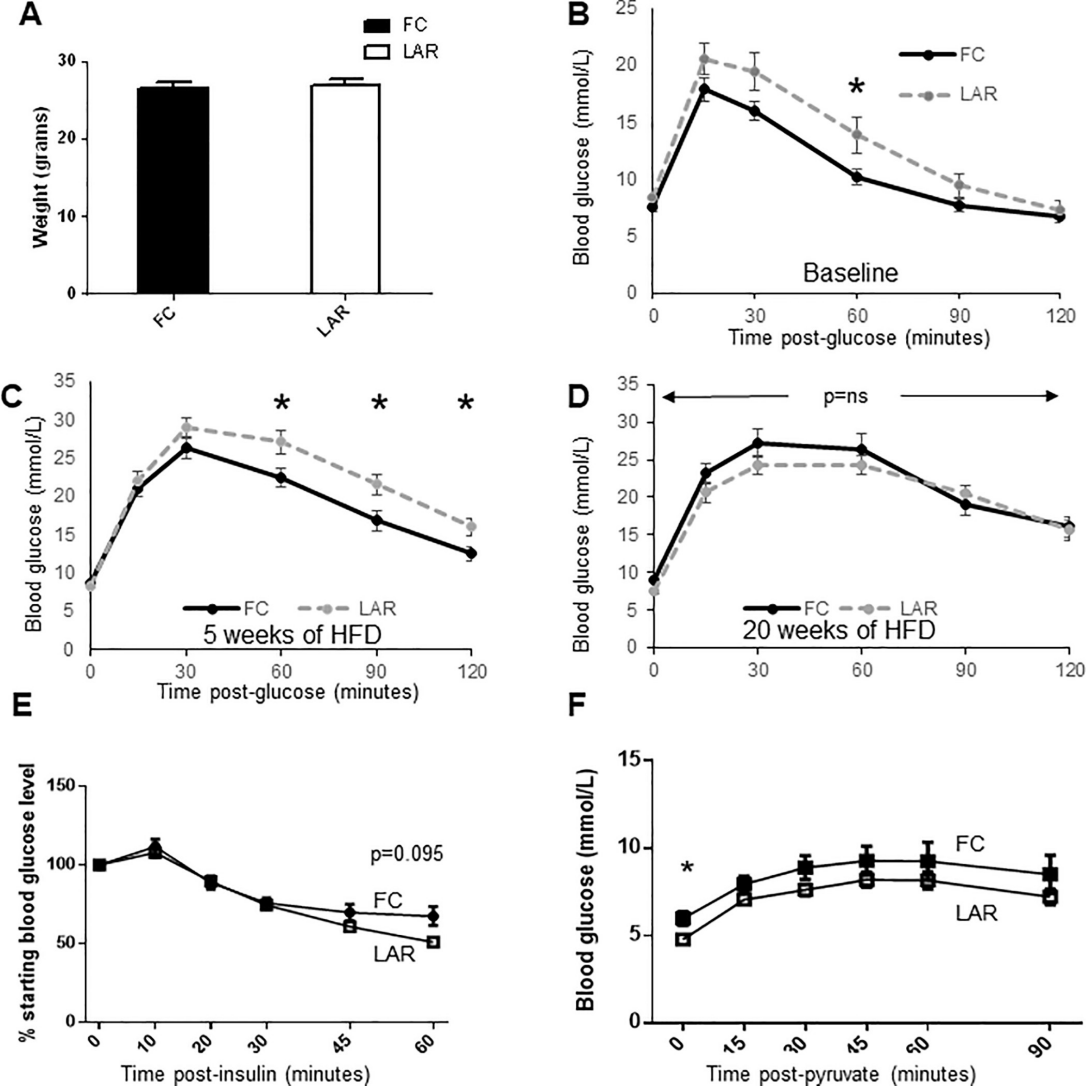
Metabolism of LAR mice. Average +/- SEM is shown. * p<0.05 by t-test and # p< 0.05 by rmANOVA. (A) Mouse weight at study commencement. (B) Glucose tolerance test in male chow-fed mice at 10 weeks of age (n = 14-16/group). (C) Male LAR and FC glucose tolerance after 5 weeks high fat diet (HFD), (n = 14-16/group). (D) Male LAR and FC glucose tolerance after 20 weeks HFD (n = 14-16/group). (E) Insulin tolerance tests for male FC and LAR mice after 11 weeks of HFD (n = 10-13/group). (F) Pyruvate challenge of male FC and LAR mice after 22 weeks of HFD (n = 8-13/group).

Prior to HFD feeding, LAR and FC mice had equivalent weight (**Fig 6A)**. As alterations in macrophage function have been shown to influence whole-body glucose metabolism [18–21], and diabetes increases risk of NAFLD / NASH, we assessed effects on glucose tolerance. Glucose tolerance tests (GTTs) were slightly worse in LAR mice at baseline, achieving significance at the 60 minute time point (**Fig 6B**, p = 0.039). Following 5 weeks of HFD feeding LAR mice had significantly worse glucose tolerance than\ FC littermates (**Fig 6C**). Glucose intolerance did not deteriorate further in floxed controls by 20 weeks and improved slightly in LAR mice, and at 20 weeks the two groups had equivalent glucose tolerance (**Fig 6D**).

The early differences in glucose tolerance were not related to differences in whole-body insulin sensitivity as assessed by insulin tolerance testing after 11 weeks of HFD (**Fig 6E**). Male LAR mice tended to have lower blood glucose concentrations at 60 minutes after insulin injection (p = 0.095). Similar results were obtained after 20 weeks (data not shown).

LAR and FC mice underwent a pyruvate tolerance test to assess endogenous glucose production. There was only a small difference in PTT results between FC and LAR mice with LAR mice having slightly but significantly lower fasting glucose **(Fig 6F)**. The incremental area under the curve did not differ significantly between groups (p = 0.43).

### Liver ARNT expression was decreased in humans with NASH

**Fig 7:**
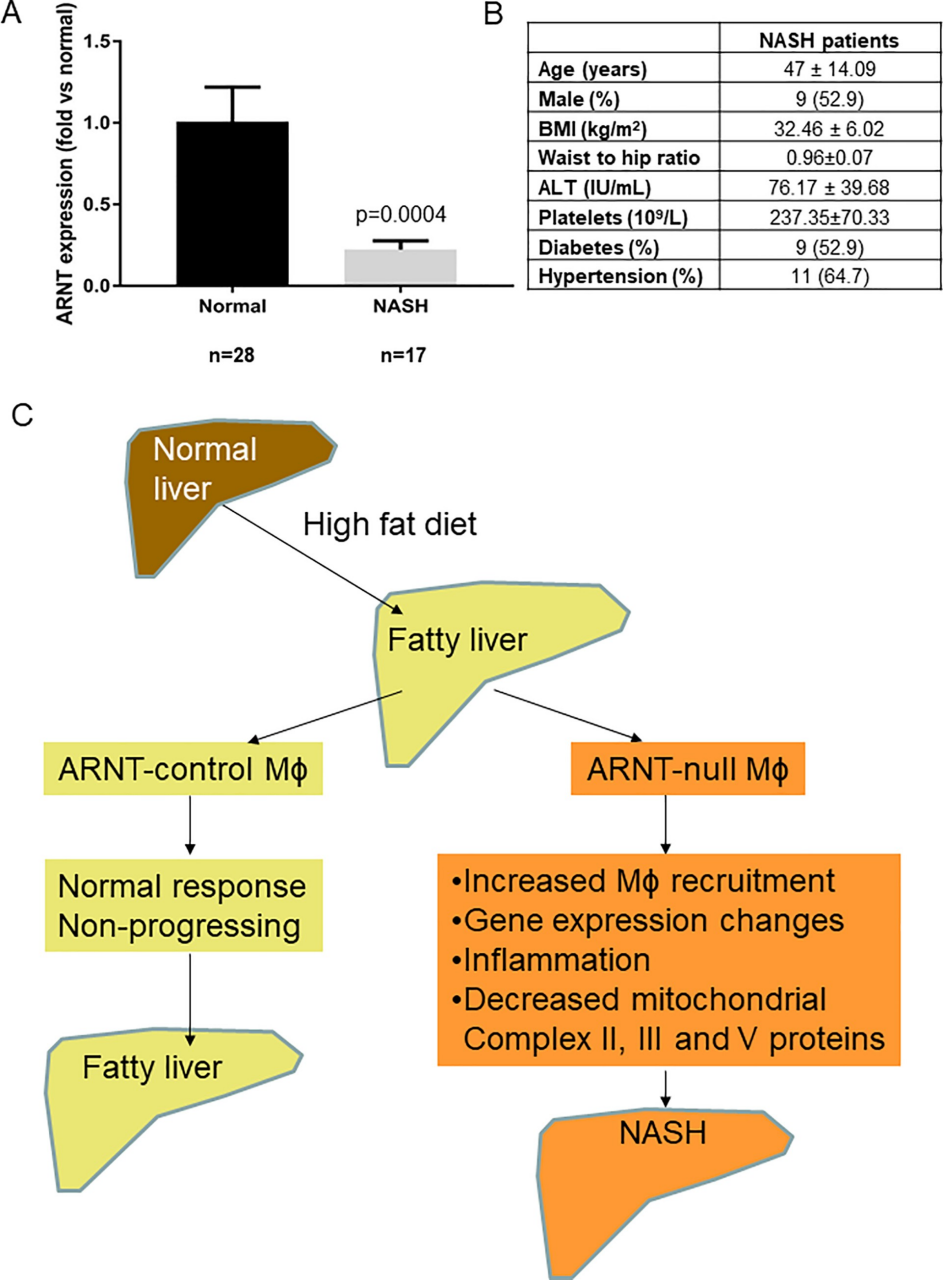
ARNT and the liver. **A)** ARNT expression in human livers from people with normal liver and with NASH. Bars indicate mean ± SEM. **B)** Patient characteristics off NASH subjects. Note data is not available for controls as the samples were de-identified. **C)** Proposed mechanisms for role of ARNT in NASH.

Finally, to examine whether ARNT might play a role in human NASH, *ARNT* mRNA was measured in liver samples from people with normal liver or NASH. *ARNT* was ~80% lower in the livers of individuals with NASH than in the livers of healthy subjects (**Fig 7**).

## Conclusions

In this study, the response of mice with myeloid cell ARNT deletion to HFD challenge was investigated. The key findings were a more than doubled prevalence of steatohepatitis (68% vs 30%), with increased lobular inflammation. This increased prevalence of steatohepatitis was accompanied by macrophage infiltration in the liver and expression of inflammatory cytokine mRNAs. No specific pro-fibrotic stimuli were given (e.g. thioacetamide, CCl4, or methionine choline deficient diet). The study used genetic controls (floxed control mice) and did not study diet-controls i.e. mice receiving normal chow. However, C57Bl/6 mice eating normal chow do not develop NAFLD. For that reason, in the ARNT-deleted mice it is not likely that there would be a strong phenotype on chow diet. Hydroxyproline was not measured to assess global liver collagen content, which is a study weakness.

Myeloid ARNT deletion increased subcutaneous fat weight, liver triglyceride content and adipocyte size and worsened glucose tolerance.

The alterations in whole body glucose / insulin homeostasis may result from changes in the liver, or adipose depots, or possibly skeletal muscle which was not studied in this work. It is plausible that there is also cross-talk between liver and fat to lead to NASH. A well-described example is adiponectin [46]. In the liver of LAR mice, Adiponectin receptor 2 had lower expression, but this was not confirmed at the protein level.

Human NASH is associated with infiltration of mononuclear phagocytes and increased liver expression of *CXCL1*, *MCP-1*, *TNF-α* and *TGF-β1* [47–50]. In mice fed a methionine/choline-deficient diet likewise, Kupffer cells play an important role in the initiation of liver inflammation, while recruited macrophages are important for disease perpetuation [51]. MCP-1 and TNF-α in particular have been shown to play important roles in the recruitment of inflammatory macrophages in NASH models, with antagonism or reduction of either associated with decreased monocyte recruitment and reduced inflammation [51–53]. Consistently, in LAR mice, increased numbers of cells of the macrophage lineage (*F4/80* mRNA expression and F4/80+ cells) were observed while the combination of increased mRNA expression for *Mcp-1*, *Cxcl-1*, *Tnf-α* and *Tgf-b1* and recruitment of macrophages appear to have perpetuated inflammation.

The finding of increased cytokine expression and inflammation in the liver of LAR mice after HFD is perhaps unexpected considering that mice lacking myeloid HIF-1α or HIF-2α have decreased inflammation in acute immune models [32–35]. Consistent with those reports, we recently reported decreased skin inflammation and wound healing in LAR animals alongside reduced cytokine mRNA in macrophages [54]. However, it has recently also been reported that mice lacking ARNT/ HIF-1α signalling in myeloid cells have increased allergic response in both a house dust mite model and an OVA murine asthma model, which may be driven by decreased IL-10 production [55]. HIF activation through myeloid cell Vhl deletion reduced inflammation in a model of chronic kidney disease, and macrophage HIF-1α deletion increased the immune response to cancer through de-repression of infiltrating cytotoxic T-cell activity [56, 57]. These results demonstrate that in certain situations myeloid cell ARNT/ HIF-1α function can reduce inflammation, but that decreased myeloid cell expression of cytokines like IL-10 may contribute to increased inflammation and NASH as observed in LAR mice. In support of this, *Il-10* mRNA was increased in LAR livers after HFD (x 2.3 fold), although not to the same extent as *Mcp-1* (x 5.2 fold) and *Tnf-α* (x 3.2 fold). In support of a potential role in humans we have found ARNT mRNA in isolated human monocytes inversely correlates with serum cytokine levels of IL-6, IL-8, MCP-1 and TNF-α [54]. There was decreased *ARNT* expression in human liver from people with NASH compared to normal liver biopsies. A limitation of the study is the lack of a fatty liver without steatohepatitis group, which we were not able to access.

There are few good mouse models of NASH. Ideally, a model should recapitulate features of the human disease with fatty liver, inflammation, perisinusoidal fibrosis, and ideally metabolic abnormalities such as obesity and increased blood glucose[58]. All of these features were observed in LAR mice fed 20 weeks of high fat diet. Excitingly, there was also a substantial decrease in expression of *ARNT* in livers of people with NASH suggesting potential human relevance of these findings. It is interesting to note that environmental toxins which activate the ARNT-partner aryl hydrocarbon receptor are associated with fatty liver disease[59, 60]. ARNT may down-regulate fatty liver disease induction by the aryl hydrocarbon receptor.

The present results add ARNT to the list of myeloid cell perturbations which result in altered metabolic function [18–21]. We note that fasting blood glucose levels were decreased at 20 weeks. This was accompanied by a trend to reduced glucose during PTT, which suggested the possibility of impaired hepatic glucose production in LAR mice. In patients with cirrhosis it has been shown that while gluconeogenesis is increased basally, glycogenolysis is decreased [61]. In addition the liver has impaired gluconeogenesis in response to gluconeogenic substrates and glucagon [61, 62]. It is possible therefore that liver dysfunction in LAR livers contributed to the decreased fasting glucose.

The proposed mechanisms are shown in Fig 7C. Lack of ARNT in macrophages leads to increased macrophage recruitment to the liver. In association with this there are changes in gene expression, including cytokines, and the proteins for mitochondrial class II, III and V are decreased. The results of this research show that deletion of ARNT in myeloid cells causes liver inflammation and steatohepatitis after high-fat diet. We also found mice lacking myeloid ARNT displayed alterations in metabolism and in fat deposition. ARNT is decreased in livers from people with type 2 diabetes, and this may contribute to their increased risk of NASH. These results suggest that myeloid cell ARNT may be a therapeutic target to reduce liver injury in patients with NAFLD and NASH.

## Abbreviations

ARNT: Aryl hydrocarbon receptor nuclear translocator
GTT: glucose tolerance test
HFD: high fat diet
HIFs: hypoxia-inducible factors
HIF-1α: Hypoxia Inducible Factor 1α
ITT: insulin tolerance test
NAFLD: Non-alcoholic fatty liver disease
NASH: non-alcoholic steatohepatitis
PTT: pyruvate tolerance test
TG: triacylglycerides T2DM = type 2 diabetes mellitus
VHL: von Hippel Lindau
